# A Bespoke Programmable Interpenetrating Elastomer Network Composite Laryngeal Stent for Expedited Paediatric Laryngotracheal Reconstruction

**DOI:** 10.1002/adfm.202513861

**Published:** 2025-11-10

**Authors:** Elizabeth F. Maughan, Lei Wu, Dong Zhang, Linxiao Wu, Graca Ferreira Vaz Pinto Mendes, Wanqiao J. Tu, Heping Ma, Toby J. Proctor, Colin R. Butler, Thomas Kjærgaard, Mark W. Lowdell, Martin A. Birchall, Robert E. Hynds, Richard Hewitt, Wenhui Song

**Affiliations:** ^1^ Department of Otorhinolaryngology Great Ormond Street Hospital NHS Foundation Trust London WC1N 3JH UK; ^2^ UCL Centre for Biomaterials in Surgical Reconstruction and Regeneration Department of Surgical Biotechnology Division of Surgery & Interventional Science University College London London NW3 2PF UK; ^3^ UCL Ear Institute London WC1X 8EE UK; ^4^ Epithelial Cell Biology in ENT Research Group Developmental Biology and Cancer Department UCL Great Ormond Street Institute of Child Health University College London London WC1N 1DZ UK; ^5^ Institute of Immunity & Transplantation University College London London NW3 2PF UK; ^6^ Department of Otorhinolaryngology Aarhus University Hospital Palle Juul‐Jensens Blvd. 99 Aarhus 8200 Denmark; ^7^ Royal National Throat Nose and Ear Hospital and University College London Hospitals NHS Foundation Trust London WC1 6JB UK

**Keywords:** 3D TIPS printing, interpenetrating elastomer network, laryngeal stent, paediatric laryngotracheal reconstruction, programmable elastomer composites

## Abstract

Laryngotracheal reconstruction surgery (LTR) is the standard treatment for paediatric upper airway stenosis, enabling tracheostomy decannulation, and restoration of vocalization. In complex infant cases, postoperative stenting is essential for graft integration and minimization of restenosis, yet available off‐the‐shelf stents are often unsuitable for small patients. Here, a case of acquired‐on‐congenital laryngeal atresia is reported, where early reconstruction in infancy was performed. To support this intervention, a personalized laryngeal stent was developed from a digitally programmable interpenetrating elastomer network (IEN) composite tailored to the patient's anatomy, balancing structural stiffness with surface softness. The stent was fabricated by combining 3D inverse‐printed thermoplastic polyurethane (3D‐TIPS) scaffolds with vacuum‐assisted silicone resin infusion, yielding an elastomer with tenable geometry, hardness, and biomechanical compliance optimized for paediatric use. Manufactured in accordance with UK Good Manufacturing Practice guidelines, the stent was deployed during LTR at 5 months of age and safely removed after 6 weeks. The stented repair healed well, with decannulation at 27 months. At 8‐year follow‐up, the patient demonstrates strong vocalization, good airway protection, and normal swallowing. Beyond this case, this work establishes a versatile platform for rapid manufacture of patient‐specific elastomer implants with programmable mechanical and surface properties for soft‐tissue reconstruction.

## Graphical abstract

A programmable interpenetrating double‐network architecture, created via 3D‐TIPS printing and resin infusion, synergistically combines thermoplastic and thermosetting elastomers to balance structural rigidity and surface softness—crucial for paediatric laryngeal stents. This patient‐specific approach enables rapid production of bespoke implants with complex geometries, supporting early tracheostomy decannulation and accelerated airway reconstruction in infants with severe laryngotracheal stenosis.

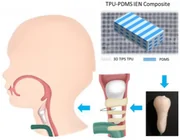

## 1 Introduction

A patent upper airway is essential for survival, and severe laryngeal or tracheal narrowing (stenosis) is immediately life‐threatening.^[^ ^1^, ^2^, ^3^ ^]^ In children, laryngotracheal stenosis most commonly arises from iatrogenic trauma due to endotracheal intubation,^[^ ^4^, ^5^ ^]^ frequently compounded by underlying congenital airway narrowing.^[^ ^4^, ^6^ ^]^ However, congenital causes alone can also lead to significant obstruction.^[^ ^7^ ^]^ While emergency tracheostomy is often lifesaving, it carries long‐term risks, including the need for continuous specialized supervision to prevent death from accidental decannulation,^[^ ^8^ ^]^ and imposes considerable psychological and logistical burdens on both the patient and their family.^[^ ^9^, ^10^ ^]^

Laryngotracheal reconstruction (LTR) is a surgical approach designed to expand a stenotic upper airway, either to obviate the need for tracheostomy or to facilitate permanent tracheostomy decannulation.^[^ ^11^ ^]^ Rather than a single intervention, LTR is best regarded as a multi‐staged process that may require repeated procedures over months or years to achieve a stable, functional airway. Many international centers have traditionally recommended delaying LTR until ≈2 years of age—allowing for reduced anaesthetic risk and the anatomical growth of larynx and cartilage donor sites. However, this wait may not be feasible in families where home tracheostomy care is impractical due to social or geographic constraints.^[^ ^4^, ^5^, ^12^ ^]^

A critical barrier to early LTR in infancy is the lack of intraluminal stents that are both appropriately sized and mechanically suited for neonates and small infants. Such devices are essential in very young, complex, or revision cases to maintain postoperative airway patency, prevent graft collapse, and minimize the risk of restenosis during healing.^[^ ^13^, ^14^ ^]^ Designing stents for this population presents unique challenges, including the need to balance extreme softness and flexibility with adequate structural integrity, particularly within the dynamic glottic inlet between the mobile vocal folds. While validated mechanical datasets for paediatric laryngeal or tracheal tissues are not yet available, adult vocal fold measurements provide a reference point, with tensile modulus as low as ≈0.03 MPa and Shore 00 hardness in the range of 1–15, reflecting their ultra‐soft nature.^[^ ^15^ ^]^ (Table S1, Supporting Information).

To address this unmet clinical need, we developed a bespoke, soft elastomer composite laryngeal stent for a 5‐month‐old infant with near‐complete laryngeal atresia of mixed (acquired‐on‐congenital) origin (**Figure** **1**). Tailored to the patient's specific anatomy and graft support requirements, the stent was fabricated from a medical‐grade composite of thermoplastic polyurethane (TPU) and polydimethylsiloxane (PDMS) using a combined 3D printing‐guided thermally induced phase separation (3D‐TIPS)^[^ ^16^, ^17^ ^]^ and silicone resin infusion process. This advanced method, compatible with UK Good Manufacturing Practice (GMP) standards, enabled precise tuning of the stent's geometry, surface hardness, and stiffness to meet complex clinical demands. Deployment of the stent enabled successful early LTR, allowing glottic patency and vocal development prior to decannulation at 27 months of age. At 8 years, the child is tracheostomy‐free with good airway, swallowing, and voice functions, underscoring the potential of patient‐specific composite stents to improve outcomes in paediatric airway reconstruction.

**Figure 1:**
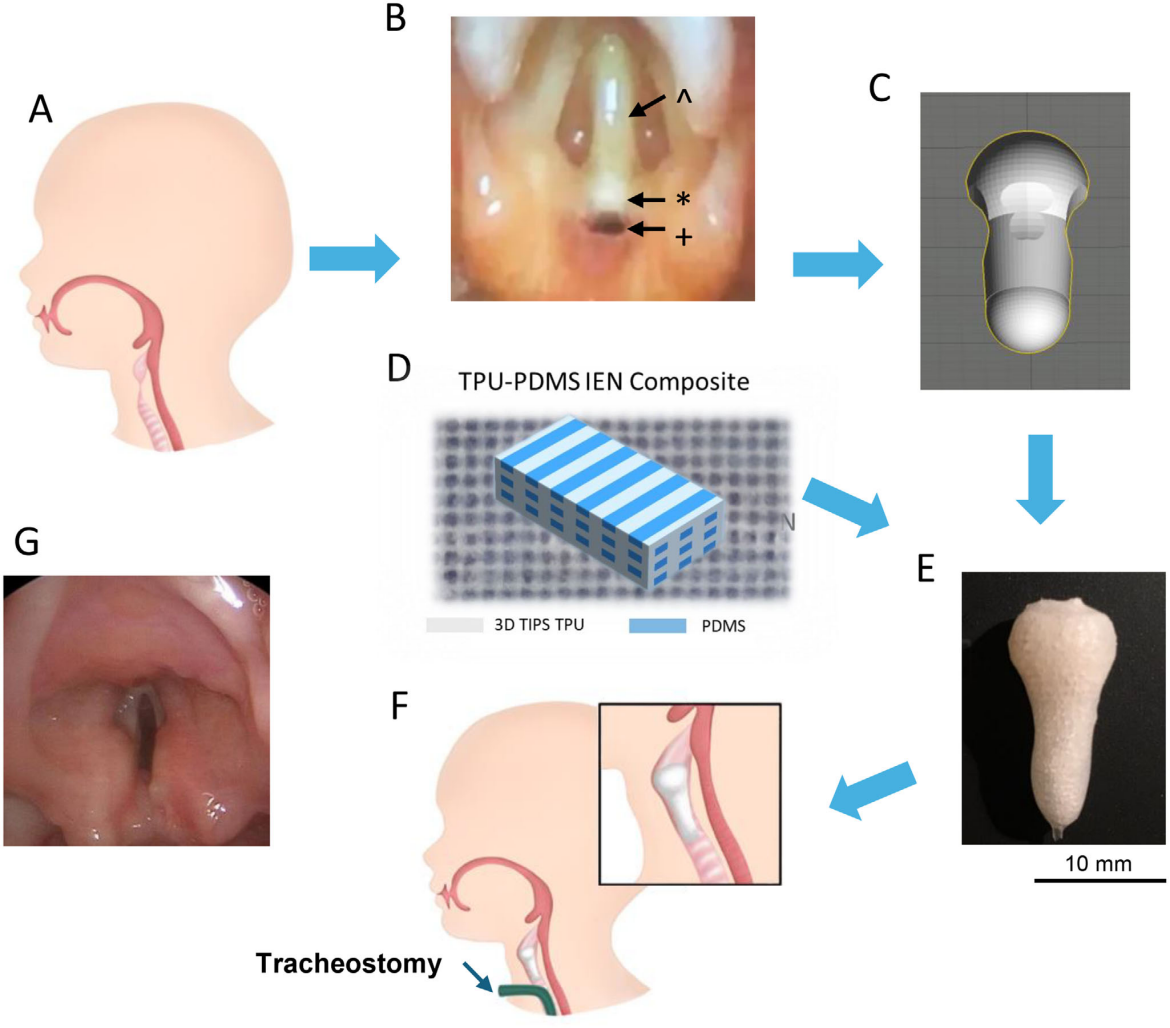
Overview of the bespoke elastomer composite stent facilitating expedited paediatric laryngotracheal reconstruction (LTR). A) Schematic illustration depicting near‐total congenital laryngeal atresia. B) Microlaryngoscopy and bronchoscopy (MLB) image at 8 weeks of age demonstrating additional acquired stenotic changes with an additional acquired element (^*^) to the congenital web‐like stenosis (^), resulting in a pinhole posterior airway (+). C) 3D design of a stent by computer‐aided design (CAD). D) Soft composite with interpenetrating elastomer networks (IEN) fabricated by combining 3D‐TIPS printed thermoplastic polyurethane scaffolds with silicone resin infusion. E) Patient‐specific soft elastomer composite laryngeal stent. F) Diagrammatic representation showing the precise anatomical positioning of the stent within the infant's larynx with a stay suture for stability during healing. G) MLB view of a normal larynx as a reference.

## 2 Clinical Case

A male neonate presented *in extremis* with immediate severe respiratory distress and stridor from the first breath, following normal delivery at 38‐week gestation at Aalborg University Hospital (Denmark) in January 2017. Dense cyanosis and oxygen desaturations developed whenever the baby was mildly agitated, which were temporarily managed via facemask continuous positive airway pressure with limited improvement. Immediate intubation encountered resistance using a size 2.0 uncuffed endotracheal tube, but was successful, and the child was urgently transferred to Aarhus University Hospital (Denmark) for further management. Microlaryngoscopy and bronchoscopy (MLB) within the first 24 h of life showed a near‐total laryngeal atresia in the glottic and subglottic regions with a pinhole posterior opening (Figure 1A,B), and therefore tracheostomy was performed concurrently. This isolated finding was confirmed by magnetic resonance imaging, which also confirmed the clinical absence of dysmorphic features or commonly associated structural abnormalities. There was no relevant family or prenatal history, and no specific fetal abnormalities had been noted on prenatal imaging, although polyhydramnios had been noted at the final prenatal ultrasound and at delivery (3–4L of amniotic fluid present). Following successful stabilization of the child's airway via tracheostomy, there were no clinical signs of aspiration via the pinhole airway, and therefore he was commenced on a bottle‐fed feeding regime with supplementation as required via nasogastric tube.

Over the following 10 weeks, the infant developed normally. A multidisciplinary discussion at Aarhus considered the long‐term management strategy. Conventional open LTR is typically deferred until ≈2 years of age to allow growth of the airway and rib cartilage for autologous grafting, and to minimize anaesthetic risk. However, this approach typically necessitates prolonged hospitalization until 24 h home tracheostomy care can be safely established. The family expressed a strong preference for exploring accelerated reconstructive options, and so the patient was referred to Great Ormond Street Hospital (GOSH), London, UK, to further evaluate toward this goal.

On MLB assessment at GOSH in March 2017, granulation tissue was found to have completely occluded the remaining laryngeal pinhole (Figure 1B), and there was an associated grade three subglottic stenosis. Of note, he also had circumferential granulations surrounding the tracheostomy stoma despite excellent stoma care. After extensive consultations with multidisciplinary airway teams and the GOSH Clinical Ethics Committee, an attempt at expedited LTR at 4–6 months of age in line with familial wishes was deemed appropriate. The goals were to create a patent upper airway for safety and for the development of voice, with planned medium‐term retention of his tracheostomy as a safety measure.

A major barrier to early surgical intervention was the absence of a suitably small, soft, and anatomically appropriate intraluminal stent to support the reconstruction during the early healing phase. Commercially available options were inadequate in terms of scale, flexibility, and conformability. To overcome this, a patient‐specific laryngeal stent was designed and fabricated using a collaborative clinical–engineering approach (Figure 1C–E). The stent was made from a medical‐grade elastomer–elastomer composite, combining TPU and silicone elastomer, and was manufactured using a combined 3D‐TIPS and silicone resin infusion process (Figure 1D). Following iterative design optimization and safety validation, the stent was produced under UK GMP conditions for compassionate clinical use (Figure 1F).

## 3 Results

### 3.1 Pre‐Implantation Evaluation of Medical‐Grade Materials

The fabrication of a patient‐specific laryngeal stent for infants presents unique material and structural challenges. The stent must accommodate a complex airway geometry and simultaneously satisfy two often opposing mechanical requirements: extreme softness for mucosal protection and sufficient stiffness for luminal support. Achieving these criteria using a single medical‐grade polymer or existing 3D printing platforms remains highly limited due to processability, filament availability, and mechanical constraints. To address this, we selected medical‐grade TPU (Carbothane AC‐4085A‐B20) and two PDMS resins with low viscosity and Shore hardness (NuSil MED‐4011 and MED4‐4420) as the base elastomers, chosen for their broad mechanical tunability, established biocompatibility, and suitability for long‐term implantation. The TPU component served as the structural “skeleton” of the stent, providing intrinsic elasticity, toughness, and puncture resistance, while the PDMS resin imparted a soft, low‐hardness interface for improved mucosal compliance and surface lubricity. Both materials are sterilizable by standard clinical protocols and have previously demonstrated clinical safety (see Table S1, Supporting Information). Leveraging the thermoplastic nature of TPU and the thermosetting behavior of PDMS, we developed digitally defined interpenetrating elastomer networks (IEN) through a combination of 3D‐TIPS and vacuum‐assisted resin infusion. This dual‐material strategy enabled spatially programmable structure–property control at both the macro and microscale.

#### 3.1.1 3D‐TIPS TPU Scaffold Fabrication and Mechanical Tuning

To enable precise structural control and mechanical tunability for paediatric airway applications, porous TPU scaffolds were fabricated using a customized 3D‐TIPS process (**Figure** **2**A). This inverse printing approach allows rapid production of complex geometries from medical‐grade or non‐printable polymers without requiring filament extrusion, which is typically infeasible for speciality materials available only in small quantities.^[^ ^16^, ^17^, ^18^ ^]^ Sacrificial polyvinyl alcohol (PVA) preforms were first 3D printed with varying infill densities (30%, 40%, 50%, and 60%) using fused deposition modelling (FDM). These infill parameters were digitally programmed to define the macro‐architecture of the final scaffold. The printed PVA structures were then infused with TPU dissolved in dimethylacetamide (DMAc), followed by immersion in deionized ice water to initiate thermally induced phase separation and coagulation of TPU. Simultaneously, the PVA template and solvent were removed, resulting in porous TPU scaffolds with final infill densities ranging from 40% to 70%. The scaffolds were thoroughly rinsed and air‐dried before characterization. Optical images of the resulting TPU scaffolds (Figure 2B) confirmed successful translation of the designed infill patterns, with porosity and channel density increasing as a function of the original PVA infill. Scanning electron microscopy (SEM) revealed highly defined interconnected macro‐porous structures at both the surface and cross‐sectional levels (Figure 2C,D), faithfully reproducing the digitally programmed architecture. Uniform micro‐ and nano‐porous structures were also observed at high resolution (Figure 2C,E), indicating a hierarchically porous structure of TPU scaffolds by 3D‐TIPS process. Mechanical testing demonstrated that scaffold stiffness, strength, and extensibility were highly dependent on infill density. Stress–strain curves revealed that all scaffolds exhibited hyperelastic behavior, like their bulk elastomer, despite their porous structures (Figure 2E). The scaffolds with higher infill density exhibited greater tensile strength and modulus, whereas lower infill density scaffolds offered increased strain. Quantitative analysis showed that tensile modulus increased with higher TPU infill (Figure 2F), while stretchability peaked at intermediate densities (Figure 2G). Ultimate tensile strength reached a maximum at 60% infill (Figure 2H), and toughness followed a similar trend (Figure 2I).

**Figure 2:**
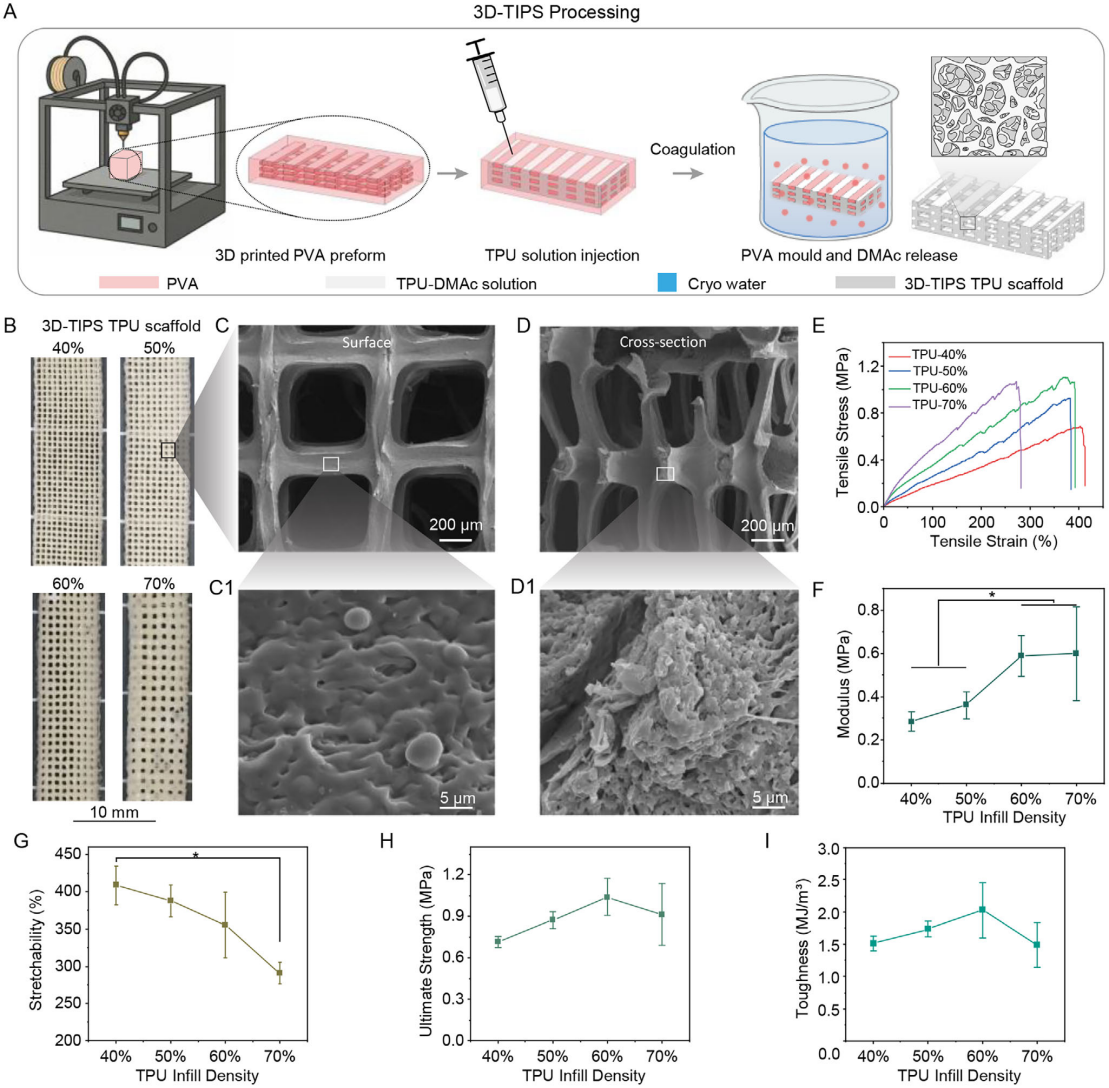
Fabrication and characterization of thermoplastic polyurethane (TPU) scaffolds via 3D printing‐guided thermally induced phase separation (3D‐TIPS). A) Schematic illustration of the 3D‐TIPS process, including 3D printing of sacrificial polyvinyl alcohol (PVA) preforms (with 30–60% infill density), infusion of TPU dissolved in dimethylacetamide (DMAc), and immersion in ice water to induce phase separation within PVA mesh mould while dissolving the PVA mould—yielding porous TPU scaffolds. B) Optical images of the resulting TPU scaffolds, corresponding to infill densities of 40%, 50%, 60%, and 70%, as determined by the original PVA template design and coagulation conditions. C,D) Scanning electron microscopy (SEM) images of scaffold surface (C) and cross‐section (D), showing interconnected and hierarchical porous architectures. E) Representative tensile stress–strain curves for TPU scaffolds fabricated at different TPU infill densities. F–I) Quantitative mechanical properties of the scaffolds as functions of TPU infill density: tensile modulus (F), stretchability (G), ultimate tensile strength (H), and toughness (I). (^*^= *p* < 0. 1).

#### 3.1.2 Fabrication and Mechanical Performance of TPU–PDMS IEN Composites

To generate a suitable stent material with sufficient stiffness and an extra soft and bioinert surface, a unique elastomer–elastomer IEN composite was designed and fabricated by utilizing silicone resin with low Shore hardness and viscosity. The dried TPU scaffolds were infused with either MED‐4011 or MED4‐4420 silicone resin via vacuum‐assisted resin infusion (**Figure** **3**A). Upon curing of the molecular crosslinking network, a macro‐scale silicone network was generated simultaneously, interpenetrating throughout the TPU interconnected network, forming an adhesive IEN composite. Optical images confirmed translucent resin diffusion across the scaffold variants for both silicone types (Figure 3B), and SEM analysis revealed a well‐defined boundary of TPU–PDMS double elastomer networks with intimate interfacial contact (Figure 3C–F).

**Figure 3:**
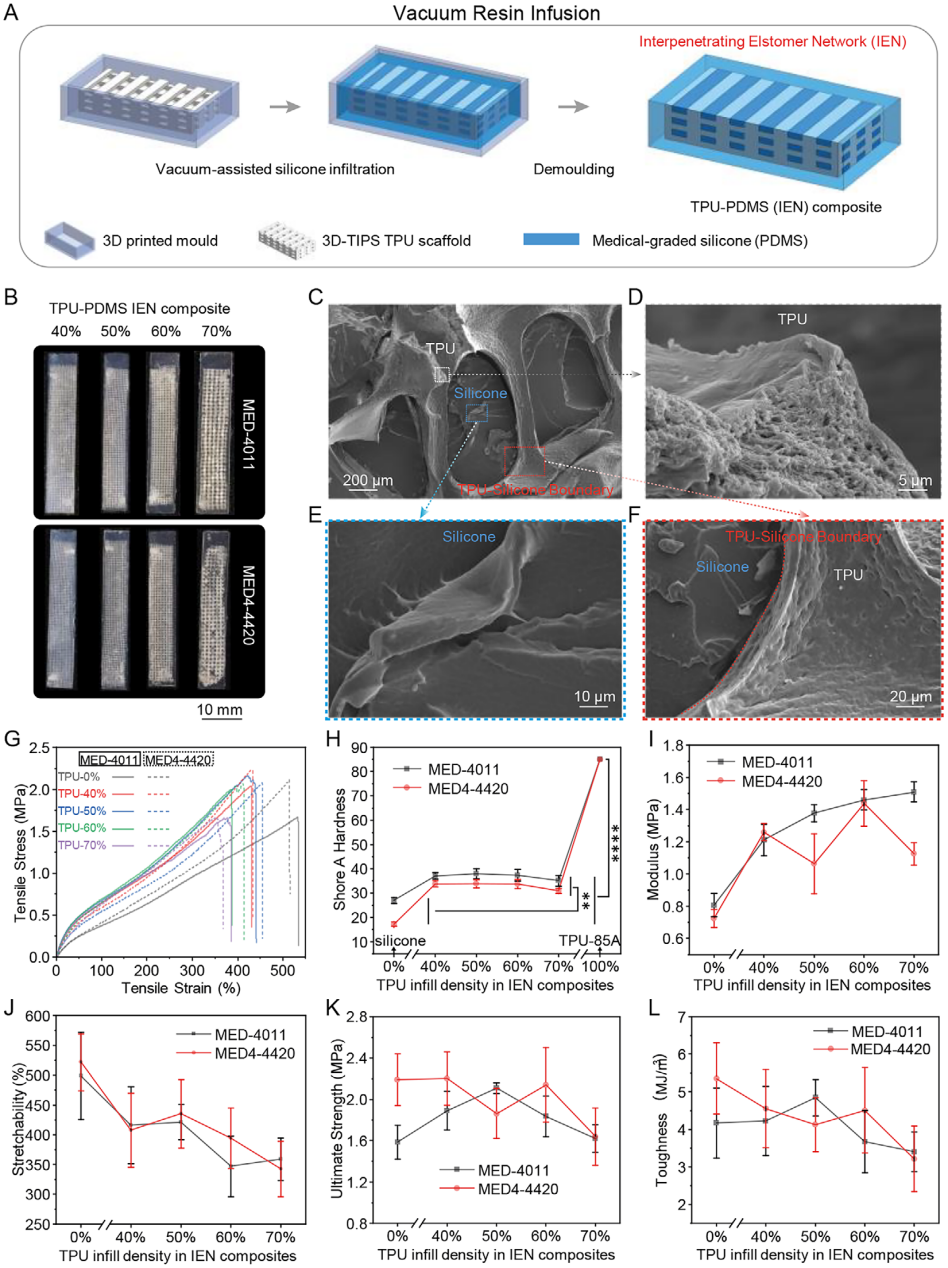
Fabrication and characterization of interpenetrating elastomer networks (IEN) composites comprising TPU and PDMS. A) Schematic diagram showing vacuum‐assisted resin infusion, in which medical‐grade silicone resin infiltrates porous TPU scaffolds produced by 3D‐TIPS, forming TPU–PDMS IEN composites. B) Photos of TPU–PDMS IEN composites prepared using two different silicone resins (MED‐4011 and MED4‐4420) and TPU scaffolds with infill densities of 40%, 50%, 60%, and 70%, respectively. C–F) SEM images demonstrating cross‐sectional fracture morphologies of the composite with TPU 50% infill and MED‐4011 silicone resin, highlighting the distinct microstructural interfaces: (C) overview of TPU–PDMS interfacial region, (D) rough, microporous fracture surface of the TPU scaffold, (E) smooth, solid fracture surface of the PDMS matrix, and (F) clear boundary between TPU and PDMS phases, indicating effective adhesion and interpenetration of the composite structure. G) Representative tensile stress–strain curves comparing mechanical behaviors across TPU scaffolds with different infill densities and PDMS types. H–L) Quantitative characterization of composite mechanical properties: tensile modulus (H), stretchability (I), ultimate tensile strength (J), and toughness (K), Shore A hardness (L), all plotted against TPU scaffold infill density for both PDMS types. (^**^= *p* < 0. 01, ^****^= *p* < 0. 0001) Note: TPU–PDMS IEN composites made from TPU scaffold with 50% infill and MED‐4011 silicone resin (C‐F) exhibited optimal mechanical performance and were therefore selected for stent fabrication in LTR surgical treatment.

As expected, the IEN composites exhibited excellent elasticity and flexibility, while varying with TPU scaffold infill density and PDMS as matrix (Figure 3G). Increasing TPU content reduced internal porosity, yielding stiffer and stronger composites. Tensile modulus ranged from 1.21 to 1.51 MPa, gradually levelling off depending on silicone type (Figure 3H), with values below those of adult thyroid/tracheal cartilage^[^ ^19^, ^20^ ^]^ (3.2–23 MPa), therefore expectedly more comparable to paediatric laryngeal tissue (Table S1, Supporting Information). The ultimate strain indicates outstanding stretchability increased with decreasing TPU infill density, ranging from 343–408% (MED‐4011) and 348–406% (MED4‐4420) (Figure 3I). Ultimate tensile strength peaked, reaching 2.11 MPa (MED‐4011) at 50% infill and 2.20 MPa (MED4‐4420) at 60% infill (Figure 3J). Toughness followed a similar trend, with maxima of 5.35 and 4.58 MJ m^−^^3^, respectively (Figure 3K). These tensile strength values fall within or exceed the mechanical envelope of adult thyroid cartilage (1–15 MPa),^[^ ^19^, ^20^ ^]^ supraglottic epiglottis (≈1.5 MPa)^[^ ^21^, ^22^, ^23^ ^]^ and vocal fold (0.35–1.75 MPa)^[^ ^15^ ^]^ (Table S1, Supporting Information). It should be noted that Shore A hardness rose from 25 (pure PDMS) to 38.0 (MED‐4011) and 17 to 33.9 (MED4‐4420) at 70% infill—both significantly softer than native TPU (Shore 85A) (Figure 3L). The composite with TPU at 50% infill density, interpenetrated by the MED‐4011 silicone network, was selected to balance structural stiffness and surface softness. It demonstrated a tensile modulus of ≈1.5 MPa, compressive stiffness of ≈1.7 N mm^−1^, and low Shore hardness (25), while allowing effective infusion capability of MED‐4011 resin during the resin‐fusion process and leveraging the proven biocompatibility of both elastomers ˗̶ making it well suited for maintaining airway patency in harmony with mucosal compliance.^[^ ^24^ ^]^

#### 3.1.3 Biocompatibility of 3D‐TIPS IEN Composites In Vivo

Cytocompatibility testing was performed using the Alamar blue assay and total DNA assay to assess the viability of human lung fibroblast cells on 3D‐TIPS TPU scaffolds and TPU‐PDMS IEN composites over 7 days (**Figure** **4**A,B). The increases of both metabolic activity and total DNA suggested low toxicity with human cells, in line with widespread uses of TPU^[^ ^25^, ^26^ ^]^ and PDMS^[^ ^27^, ^28^ ^]^ elastomers in medical devices.

**Figure 4:**
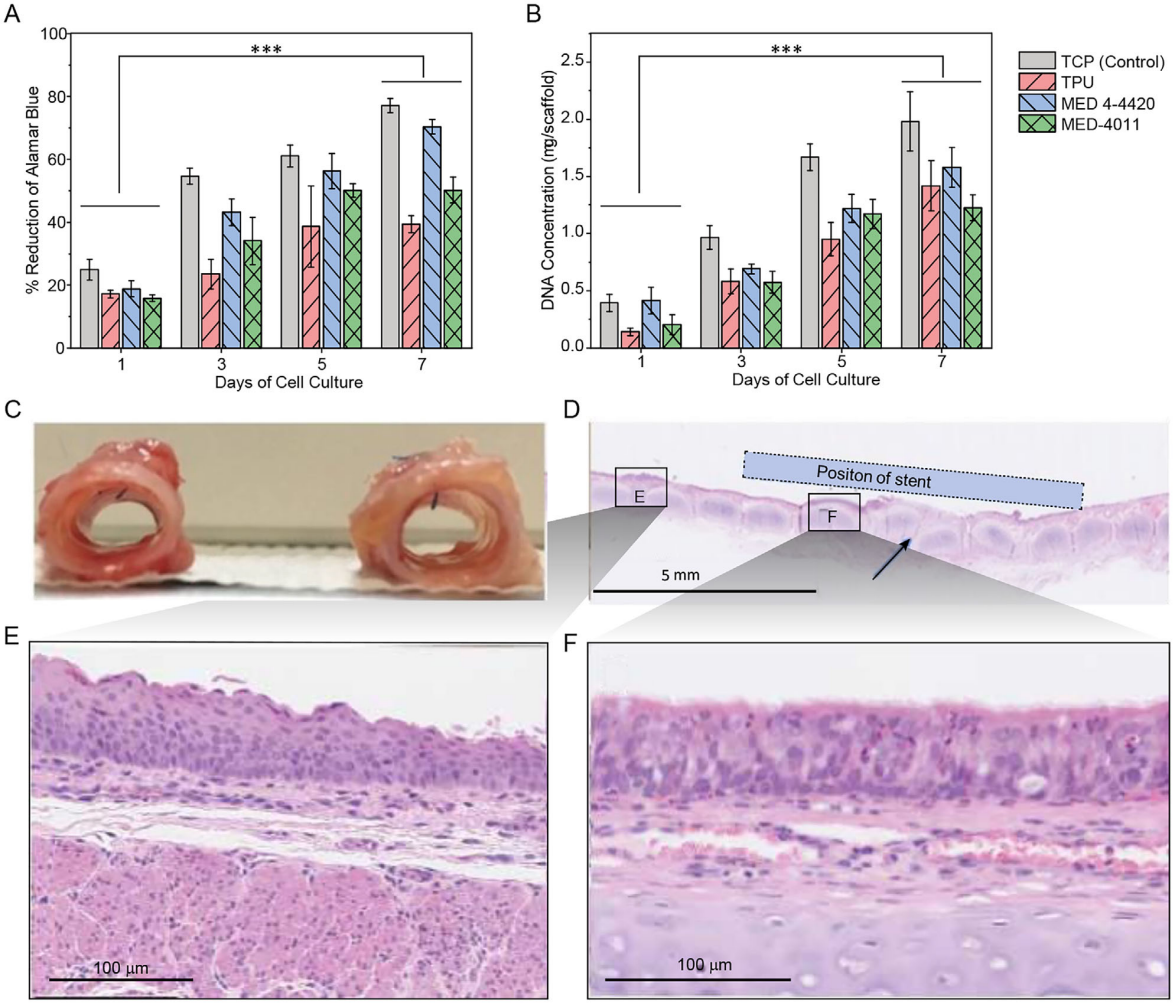
In vitro and in vivo biocompatibility testing. A) Metabolic activity and B) total DNA analysis of human lung fibroblasts on 3D‐TIPS TPU scaffolds and TPU‐PDMS IEN composites (two types of silicone resin MED‐4011 and MED‐4‐4420) over 7 days. Tissue culture plastic (TCP) served as a positive control, n=6. (^***^= *p* <0. 001) C–F) Airway in vivo biocompatibility testing of MED‐4011 PDMS/TPU IEN composites. (C) Explanted rabbit tracheae showed no gross macroscopic or microscopic differences in lumen calibre in the stented areas. (D) No granulation‐based changes were seen associated with the stented area. The blue arrow denotes the area of the primarily re‐sutured tracheotomy for stent insertion. (E) Epithelium in contact with the distal end of the stent (most susceptible to friction) showed some evidence of stratified squamous metaplasia, but areas beneath the main body of the stent remained of a healthy pseudostratified ciliated morphology (F).

Following transplantation of IEN composite tubes in rabbits (*n* = 4), none of the animals exhibited any postoperative complications during the two‐week monitoring period, and all reached the termination date without any clinical signs of breathing difficulties. Upon termination, macroscopic examination revealed patent airways free from stenosis or granulation in either IEN composite stent or endotracheal tube controls, and all stents were removed without difficulty, i.e., no tissue integration had occurred (Figure 4C). Histological analysis, performed in a blinded manner, showed localized acute inflammation at the tracheal surgical site, with inflammation gradually diminishing toward normal tissue along the stented region (Figure 4D). There was no evidence of collapse on explanation and no evidence of cartilage fragmentation or significant inflammatory infiltrate in the stented region. Mild fibrosis with squamous metaplastic epithelium was observed at the stent edges (Figure 4E), a common observation in the clinical scenario due to small repetitive movements of the stent end against the tracheal wall with respiration and swallowing, while the overlying epithelium in contact with the stent body remained histologically normal (i.e., pseudostratified; Figure 4F).

### 3.2 Laryngeal IEN‐Stent Design, Manufacture, and Characterization

The custom stent was designed using computer‐aided design (CAD) based on measurements taken during MLB to match achievable target neolaryngeal dimensions (**Figure** **5**A). A PVA preform for the fabrication of a laryngeal stent scaffold was then 3D‐printed with a 50% infill density. The stent scaffold structure was created by injecting a TPU solution (LUBRIZOL AC‐4085A Carbothane, DMS, FDA‐approved medical grade) into the preform mould and processing it with cryo‐coagulation (3D‐TIPS), resulting in a porous TPU framework. Silicone resin (MED‐4011, Dow Corning, FDA medical grade) was then infused into this TPU scaffold using a resin infusion method, forming the final IEN composite laryngeal stent, following packaging and sterilization (Figure 5A). The mechanical properties of the 3D‐TIPS TPU stent scaffold and the IEN composite stent were evaluated through both static and dynamic compression tests (Figure 5B–D). The IEN composite stent displayed a significantly higher compressive stiffness (≈1.7 N mm^−1^) compared to the TPU stent scaffold alone (≈0.11 N mm^−1^) (Figure 5B). Under a 1 Hz sinusoidal compressive displacement of 1 mm, the IEN composite stent maintained consistent compression force over 20 000 compression cycles, with no notable force drop (Figure 5C), which is highly relevant for a stent placed within a larynx with mobile vocal cords. Furthermore, the stent showed low hysteresis throughout the testing (Figure 5D), indicating stable mechanical performance and hyperelasticity with little energy loss. Following verification of the composite stent's mechanical performance and favorable in vivo airway biocompatibility, approval for a non‐CE‐marked custom design and stent fabrication process was obtained from the UK Medicines and Healthcare products Regulatory Agency on humanitarian grounds. Additionally, the GOSH Clinical Ethics Committee granted a favorable opinion for its use.

**Figure 5:**
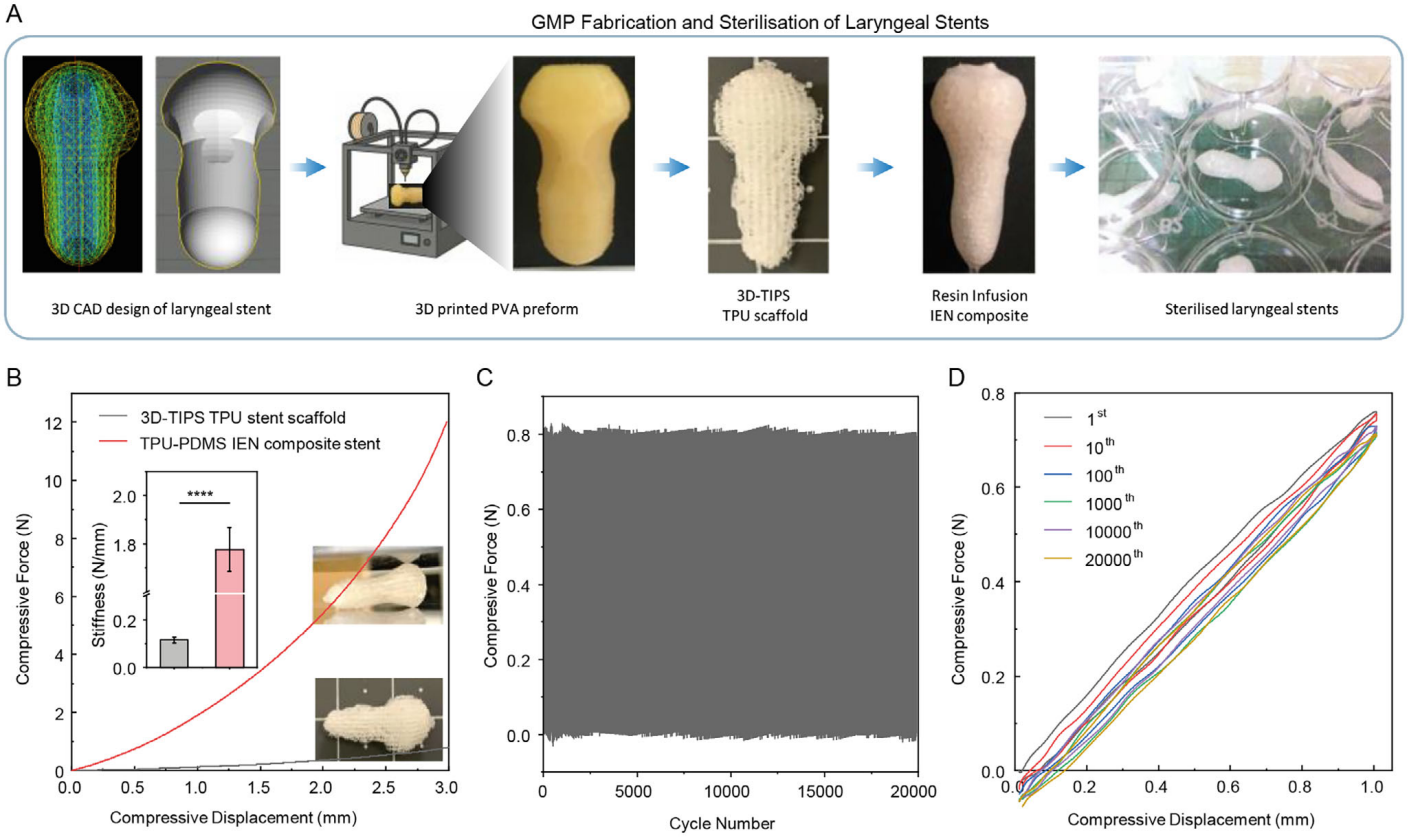
Design, GMP‐compliant fabrication, sterilization, and mechanical characterization of a bespoke paediatric laryngeal stent. A) Workflow illustrating stent fabrication steps: 3D computer‐aided design (CAD), 3D printing of a PVA preform, formation of a TPU stent scaffold via 3D‐TIPS processing, silicone resin infusion to create a TPU–PDMS IEN composite stent, and final sterilization for clinical use. B) Compressive force–displacement curves comparing the mechanical behavior of the initial TPU scaffold and the final TPU–PDMS IEN composite stent. The inset shows significantly enhanced stiffness of the composite (^****^= *p* < 0.0001) with corresponding images under compressive loading. C) Compressive force profile demonstrating mechanical stability over 20 000 cyclic compression tests. D) Overlay of compressive force–displacement curves at selected intervals (1st–20 000th cycles), confirming consistent mechanical performance, durability, and resilience under repeated loading.

### 3.3 Clinical Use

#### 3.3.1 Clinical Implantation

With full parental informed consent, a single‐stage laryngotracheal reconstruction and placement of the custom‐designed laryngeal stent were conducted on single‐patient compassionate grounds at GOSH in June 2017 (**Figure** **6**). The vocal cord atresia was endoscopically divided along the midline, followed by reshaping of the cords using cold steel techniques (Figure 6A,B). The grossly abnormal laryngotracheal skeleton was then exposed, and the thickened cricoid cartilage was carefully divided along the midline. To widen the subglottis, an anterior costal cartilage graft was placed. The custom stent was positioned endoscopically (Figure 6C), and the stent was secured by the gold standard clinical technique of a through‐and‐through “stay” suture through the airway and stent with subcutaneous burying of the suture knot outside of the airway. At the conclusion of the procedure, on‐table bronchoscopy through both the mouth and tracheostomy confirmed the stent was well‐fitted and stable within the supraglottis and glottic inlet (Figure 6D,E), and of appropriate length to ensure glottic and subglottic airway support and structural integrity during post‐surgical healing.

**Figure 6:**
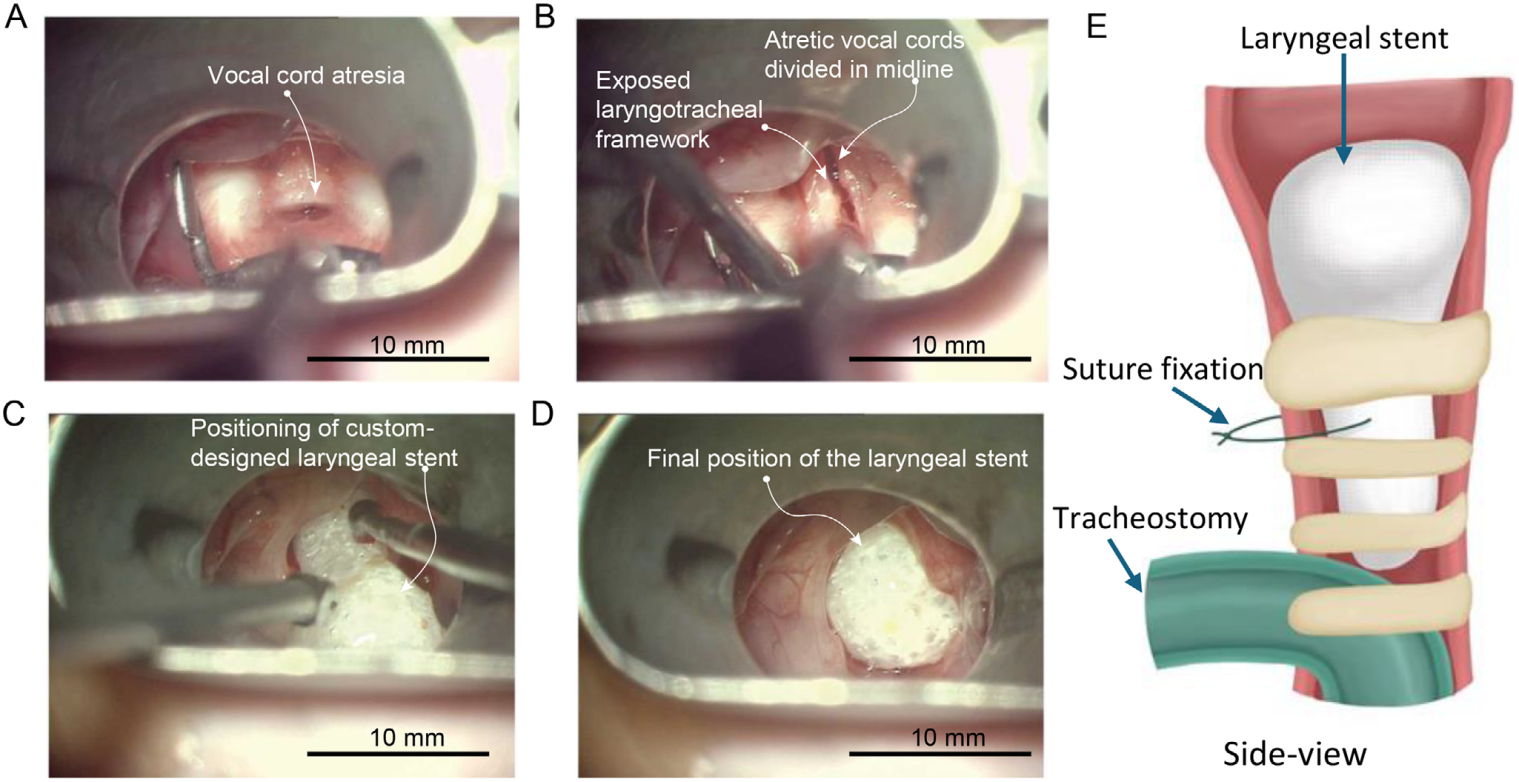
Intraoperative clinical photographs during single‐stage laryngotracheal reconstruction and laryngeal stent placement. A) Endoscopic view showing the vocal cord atresia divided along the midline. B) Reshaping of the vocal cords and exposure of the laryngotracheal skeleton. C) Positioning of the custom‐designed laryngeal stent following placement of an anterior costal cartilage graft. D) View of the position of the stent within the laryngeal inlet, and E) Schematic side‐view of securement with stay sutures for stability during healing.

#### 3.3.2 Postoperative Course

The patient recovered well from the reconstructive procedure, in line with the standard of clinical care. He recovered immediately after the procedure in a High Dependency Unit setting and was transferred to the airway ward once comfortable and breathing well through his prior tracheostomy. Regular nebulized saline and suction were delivered via his tracheostomy to help clear any blood collected during surgery, and he suffered no respiratory distress at any point. He tolerated the stent well, with only moderate difficulty swallowing. He was discharged to his referring center following a re‐check MLB on Day 7 post‐stent removal and remained an inpatient there for tracheostomy care and help with oral intake modifications. Due to practical and logistical reasons, scheduled post‐operative bronchoscopy and stent removal occurred 6 weeks post‐surgery (**Figure** **7**A). Briefly, the stay suture knot was identified by local dissection in the subcutaneous tissues of the neck, cut, and removed, allowing for the stent to be slid out endoscopically (Figure 7B). Inspection of the newly widened airway revealed a well‐maintained neolaryngeal and subglottic calibre (Figure 7C), though early granulations were present at both the surgical and tracheostomy sites, which were endoscopically treated as per clinical standard of care. Importantly, coordinated bilateral movement of freely mobile arytenoids was observed with both inspiration and expiration, a good prognostic sign for vocal cord function. Early assessment of speech and swallowing showed some vocalization effort and an intact swallow for liquids and purees, with no signs of aspiration. Following surgery, the patient remained under close observation at his referring center, with regular returns for endoscopic evaluation to monitor neolaryngeal healing and provide any necessary balloon dilation (Figure 7D–F). This proactive care, aligned with standard practice for a two‐stage LTR process, addressed a pronounced individual tendency for granulation tissue formation in the upper trachea distal to where the stent had been deployed, particularly around the tracheostomy site. Recurrent suprastomal granulomas, triggered by minimal trauma, significantly delayed tracheostomy decannulation (Figure 7G). 18 months post‐surgery, a moderate degree of stenosis in the subglottis (below the level of the previous repair) required further airway augmentation with an anterior rib graft (Figure 7H), which healed successfully with periodic early dilatations (i.e., without the need for additional stent support). His tracheostomy was decannulated in April 2019 at the age of 27 months, and no further airway dilatations have been required since March 2018. The patient has subsequently been diagnosed with 22q11 deletion‐syndrome and has some minor cognitive setbacks. At the age of eight years, he has a stable airway and exhibits effective laryngeal function, including normal swallowing, airway protection, and strong voice production.

**Figure 7:**
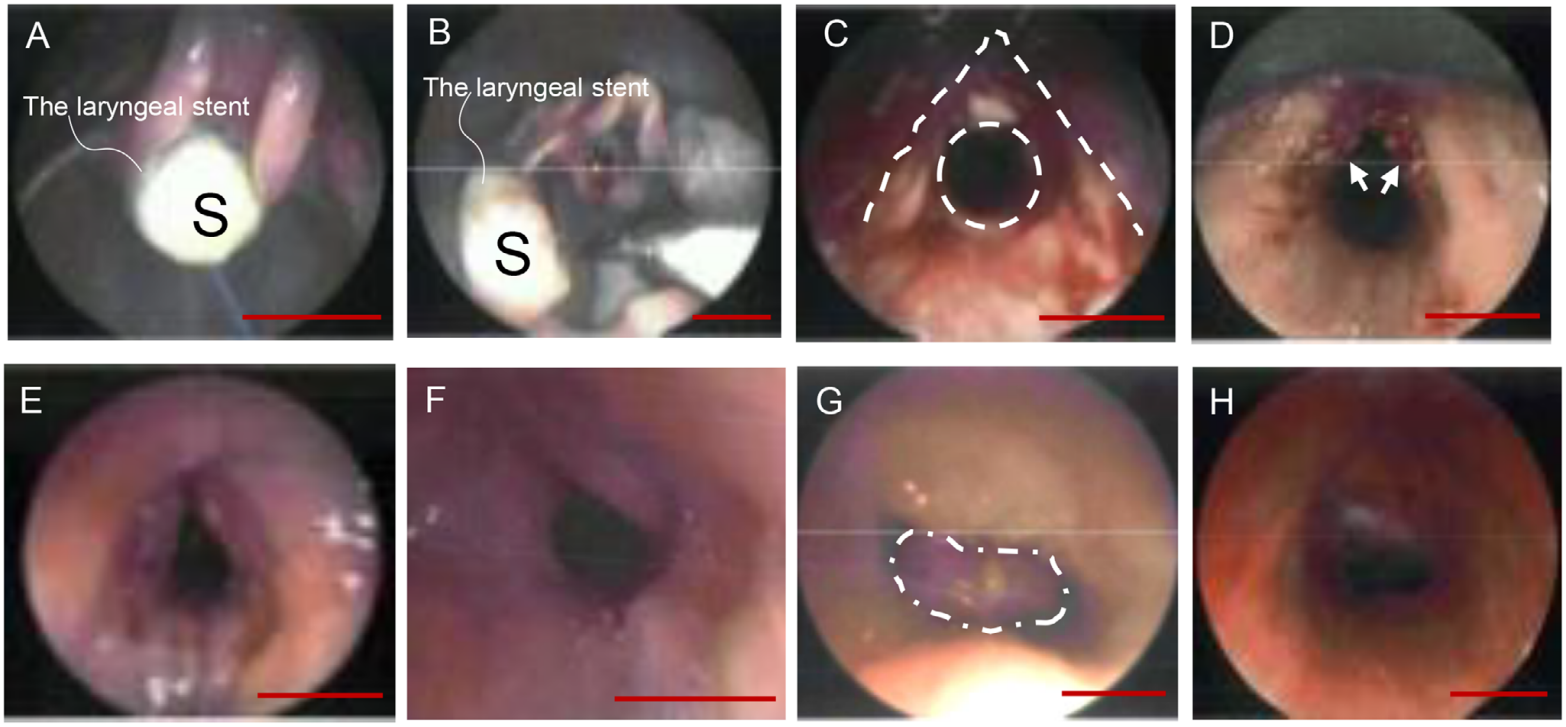
Follow‐up clinical photographs of endoscopic stent removal and treatment. A) The laryngeal stent was visualized in the correct position after 6 weeks, immediately prior to removal. B) The stent (S) was removed endoscopically following cutting of the stay suture holding in place via a small anterior neck incision, revealing a patent glottis and subglottis without immediate granulations at this level C). Endoscopic views of the neo larynx D) 10 days, E) 3 months, and F) 2 years following glottic reconstruction showed stable patency of the glottis following stent removal showing expected granulation tissue over vocal cord edges. G) The patient displayed a tendency to form granulations, as evidenced by his recurrent suprastomal tracheostomy‐associated granuloma distal to the reconstruction. Treatment of this by further resection and expansion of the area with further costal grafting H) was necessary to allow for tracheostomy decannulation, though he was able to voice via the glottis throughout this time. Scale bar: 10 mm.

## 4 Discussion

Airway injuries from traumatic intubation frequently heal with scar contracture, which will cause stenosis in circumferential defects. Advances in neonatal and paediatric intensive care have increased survival rates for critically ill and premature infants, many of whom develop complex laryngotracheal conditions that necessitate tracheostomy to maintain passage of air by bypassing the larynx.^[^ ^29^ ^]^ Congenital laryngeal atresia, web or near‐total stenosis, is extremely rare and can be associated with underlying syndromes such as 22q‐11 deletion syndrome,^[^ ^6^, ^7^, ^30^, ^31^ ^]^ occurring due to failures of laryngeal epithelial recanalization around week 10 of gestation. If obstruction is total, fluid accumulates in the developing lungs, resulting in congestive heart failure and fetal oedema^[^ ^32^, ^33^ ^]^ that can be detected on routine obstetric ultrasound screening assessment, leading to a prenatal diagnosis^[^ ^34^ ^]^ and the ability to plan for a high‐risk delivery.^[^ ^35^ ^]^ However, in cases with a small airway opening that allows fluid escape to decompress the lungs, the condition may go undetected in utero or with a diagnosis of polyhydramnios (from delayed fetal swallow), until immediate respiratory distress at birth, a fatal scenario without the immediate intervention that was successful in this case.

Managing a tracheostomy requires constant vigilance and meticulous care to clear airway secretions. There is a well‐documented risk of tracheostomy blockage or accidental dislodgement leading directly to the child's death.^[^ ^36^, ^37^ ^]^ These risks are particularly severe when there is no patent airway above the tracheostomy, preventing emergency oxygenation or ventilation, a stark contrast to tracheostomies performed for other medical reasons, where the upper airway remains accessible for intubation in the event of decannulation emergencies. This is akin to the “no upper airway” concerns associated with laryngectomy, a nationally recognized cause of significant patient harm in the UK as well as other countries,^[^ ^38^ ^]^ though there are no formal national guidelines for the paediatric context. The necessity for continuous observation, often placing a significant burden on trained parents at home, may require prolonged hospital stays until reconstruction, depending on the availability of community paediatric tracheostomy services, which vary widely by geographical region.

LTR involves endoscopic and/or open surgery to split the laryngeal cartilage framework and insert highly individualized cartilage grafts (carved from autologous rib) as spacers.^[^ ^39^ ^]^ Standard international practice generally defers LTR until ≈2 years of age or a weight of 15 kg. However, the child remains entirely dependent on their tracheostomy until that surgery can take place. An expedited reconstruction process was considered in this child as part of a family‐centered approach. The GOSH clinical ethics committee considered that, even if the expedited approach did not achieve its goal of early tracheostomy decannulation, establishing some degree of upper airway patency would significantly reduce the mortality risk associated with his tracheostomy.

A major obstacle to performing LTR at a young age was the lack of a suitable laryngeal stent to support the repair. Postoperative stenting was felt to be crucial for this patient, as in other challenging cases, to reduce re‐narrowing of the reconstructed larynx from circumferential scarring and to support any spacer cartilage grafts placed during surgery. The ideal LTR stent should be soft, flexible, and anatomically contoured to fit an individual's larynx, thereby optimally supporting grafted areas and minimizing mucosal pressure points, for example, with vocal cord movement during coughing, swallowing, and attempts to vocalize.^[^ ^40^ ^]^ This is particularly relevant for congenital surgery where neovocal cords are fashioned, as the anterior commissure of the glottis is critical for voice production and highly susceptible to scarring.^[^ ^11^, ^12^, ^41^ ^]^

At the time of treatment, there were two commercially available laryngeal stents (Figure S1, Supporting Information). The LT‐Mold (Switzerland) is a rigid silicone stent produced by casting moulds from cadaver larynges^[^ ^27^ ^]^ (with a high hardness shore value of A 50). Though these have been used successfully in children,^[^ ^42^, ^43^ ^]^ the available standardized sizes were unsuitable for our infant patient.^[^ ^31^ ^]^ The alternative Rutter Stent is designed for subglottic and tracheal use rather than use in the larynx, is circular in cross‐section, and was deemed too rigid. There was also concern that suboptimal support of key laryngeal contours would cause posterior vocal cord pressure necrosis, granulation formation, and anterior commissure scarring and deformity,^[^ ^44^, ^45^ ^]^ especially in very young or delicate airways. Both stents posed additional logistical concerns in our healthcare setting related to cost and supply availability. Standard rigid silicone endotracheal tubes are often used as stents in straightforward LTR procedures, but these are also rigid and circular in cross‐section.

To address these challenges, we developed a custom‐designed, 3D‐printed IEN composite stent by leveraging the material properties and processability of medical‐grade thermoplastic TPU and thermosetting PDMS, using an in‐house developed 3D‐TIPS additive manufacturing process. These two families of elastomers are widely used in biomedical devices due to their broad range of hyperelasticity, strength, toughness, hardness, and excellent biocompatibility. Both materials are suitable for long‐term implantation, can be sterilized using standard protocols, and demonstrated airway tissue‐specific biocompatibility in both in vitro and in vivo evaluations. 3D‐printing for airway stents is increasingly reported in the medical literature,^[^ ^46^ ^]^ the majority of which are aimed at the trachea and bronchi. The biomechanics of the larynx require specialized considerations regarding softness and elasticity, given that the moving vocal cords are likely to rub against the material with every respiratory cycle and attempt at phonation.

Despite their shared hyperelastic characteristics, TPU and PDMS differ significantly in fatigue resistance, fracture behavior, and processability, owing to their distinct macromolecular chain structures. TPUs, composed of highly entangled linear chains, exhibit superior resistance to fatigue, wear, and puncture. This is largely due to their nanophase‐separated structure, where hard‐segment domains serve as physical crosslinkers within the soft‐segment matrix. As a result, TPU is often used in applications requiring high durability and puncture resistance, a desirable quality clinically to prevent tearing of the stent when sutured into place. Additionally, TPU is readily processable using conventional injection moulding and extrusion techniques for mass production, and it can be directly printed using FDM printers—provided printable filaments are available. However, medical‐grade TPU filaments were not commercially available, and even custom‐made filaments present challenges in printing soft TPUs with Shore hardness below 80.

In contrast, medical‐grade PDMS, with its lower Shore hardness, is better suited for creating the soft surfaces required in paediatric laryngeal stents. Although its low‐viscosity siloxane resin allows for conventional moulding and infusion processes, it is not suitable for direct 3D printing. Moreover, PDMS forms a covalently crosslinked 3D network upon curing, which, while beneficial for stability, makes it prone to tearing when subjected to stress or suturing.

We employed 3D‐TIPS to print TPU scaffolds in reverse, forming a highly hierarchical and interconnected porous structure. This structure comprises a combination of digitally defined macropores and 3D‐TIPS induced micro‐/nano‐pores generated through phase separation during coagulation, within a confined PVA mesh, serve as a sacrificial mould. This design enhanced the scaffold's compliance and substantially increased the intra‐scaffold surface area, facilitating uniform infusion of the silicone resin throughout the TPU network and adhesion between the two elastomers (Figure 2B–D). Following this, the scaffold was infused with silicone resin to create TPU‐PDMS IEN composites. This resulted in a unique architecture consisting of interpenetrating thermoplastic and thermosetting double rubbery networks (Figure 3B–D). The crosslinked silicone network became tightly and regularly entangled within the micro‐/nano‐porous TPU network, contributing to the composite's cohesive hyperelasticity and mechanical durability, while simultaneously providing an extra‐soft surface. Importantly, the architecture and ratio of TPU to PDMS in these interconnected networks are digitally programmable and highly reproducible. This enables the development of a new class of elastomer composites with a broad, tunable range of mechanical properties and surface hardness.

The ability to customize the stent's stiffness and softness by adjusting the TPU scaffold's infill density and selecting different types of silicone resin enabled precise tuning of the composite's modulus and stretchability. This allowed us to achieve an optimal balance between rigidity and flexibility−critical for the delicate paediatric larynx. This final stent was fabricated using an optimized combination of 50% TPU infill density and MED‐4011 silicone resin (Shore hardness 25). The resulting IEN architecture provides the desired surface softness and elasticity to match patient's specific anatomical needs, while retaining the excellent fatigue and tear resistance inherent to TPU. Notably, the stress–strain behavior of the composite stent within 20% strain closely matched that of human tracheal cartilage,^[^ ^47^ ^]^ suggesting that the stent can offer adequate structural support to maintain airway patency while preserving the flexibility needed to prevent localized pressure or irritation in the laryngeal region. The risk of tissue adhesion should be considered for any stent material designed for long‐term indwelling. Tracheostomies and silicone stents on the commercial market (e.g., Montgomery, Rutter) do not clinically lead to tissue adhesion. Together with the favorable cytocompatibility and short‐term in vivo airway response (Figure 4), these findings indicate that the IEN approach provides a materials platform capable of balancing luminal support with mucosal compliance and is transferable to related soft‐tissue interface devices.

Our manufacturing approach, combining 3D‐TIPS printing with resin infusion, enabled the rapid and precise fabrication of devices with complex geometries and internal architectures using only the necessary quantity of medical‐grade materials. This process supports on‐demand, patient‐specific production within clinically practical timeframes, potentially eliminating the advantages of off‐the‐shelf commercial stents. Moreover, the method and materials are cost‐effective and adaptable, and this single patient use demonstrates the potential of this technology to enable treatment for other individual complex patients whose anatomical requirements are not fulfilled by existing commercially available stents. It would also support targeted laryngeal drug‐eluting strategies to aid local healing.^[^ ^48^ ^]^

The custom soft stent remained in place for six weeks, during which time the airway inlet healed around it. On in vivo modelling, squamous metaplasia was observed at the edges of the stent (i.e., at the points of maximal friction between stent and airway). In health, the vocal cords are covered by stratified squamous epithelium and increased growth of this can occur with repeated trauma (as in Singer's nodules). Clinically, these settle with removal of the traumatic stimulus and we would therefore anticipate the same to occur on stent removal. Upon removal, granulation tissue was observed around the stent, which is a common finding following this type of reconstruction. The stent's soft structure allowed for minimal additional trauma to the neolarynx during the healing period, thereby minimizing the degree of fibrosis/restenosis caused by scarring, which could hamper glottic calibre with growth. The patient responded well to standard postoperative management, including gentle removal of granulation tissue and balloon dilations. He required additional surgery to the subglottis and upper trachea 18 months after the initial procedure, to address recurrent granulations and stenosis from trauma associated with previous intubation and tracheostomy. His propensity for granulation growth in the region of his tracheostomy was the major determinant in his decannulation decision‐making.

Prolonged stenting may rarely induce tracheomalacia, particularly when inflammation is provoked by trauma or infection.^[^ ^49^ ^]^ Our in vivo studies were designed to run for two weeks, which is considerably less time than would be clinically anticipated to cause risk of malacia. Clinically, the patient incurred no laryngeal malacia and underwent no further surgery in the stented region, but rather underwent further expansion surgery below. It would not have been ethical to re‐biopsy the patient's reconstruction without clinical grounds, so no resection samples exist containing cartilage from the patient on which to perform research histological or biochemical testing.

At 8 years of age, the patient is tracheostomy‐free and demonstrates excellent laryngeal function across voice production, airway protection, and swallowing domains, without the need for additional airway endoscopic procedures since February 2021. His tracheostomy was decannulated in April 2019 at the age of 27 months, which compares favorably with the likely timeline of decannulation had he followed a conventional second‐stage LTR process.  This decannulation allowed for the development of voice‐based speech and language in line with his peers. The presence of an upper airway lumen following surgery to the time of decannulation significantly reduced his personal risk of mortality in the event of accidental decannulation. This first‐in‐infant case study demonstrates feasibility but requires further validation in larger cohorts.

Beyond the paediatric laryngeal stent demonstrated in this study, the digitally programmable IEN composites developed here represent a versatile platform for the design and fabrication of patient‐specific elastomer composite materials and soft implants, enabling translation from bench to bedside within a short lead time—a capability not previously described. By integrating 3D inverse printing with vacuum‐assisted resin infusion, this strategy allows independent and quantitative control of scaffold porosity, stiffness, interfacial morphology, and surface hardness, thereby producing mechanically adaptive elastomer composites and implants tailored to diverse soft‐tissue environments. The TPU scaffold further reinforces the structure, which is anticipated a significant enhancement of suture retention and fatigue resistance strength compared with silicone alone. These tuneable structural–mechanical properties are broadly relevant to biomedical devices such as tracheal and oesophageal stents, vascular grafts, auricular and nasal cartilage supports, and customised soft‐tissue scaffolds. Moreover, the platform's ability to generate complex geometries and controlled mechanical gradients for patient‐specific needs underscores its potential to accelerate the clinical translation of functional elastomers across multiple indications.

## 5 Conclusion

We have demonstrated a tailored, multidisciplinary, patient‐centered approach to accelerate laryngotracheal reconstruction in early infancy through the development of a patient‐specific stent. By combining 3D‐TIPS printing with resin infusion techniques, we created a new class of TPU/silicone composites featuring a wide range of tuneable hyperelasticity, stiffness, and surface hardness. The programmable interpenetrating double‐network architecture synergistically leverages the hyperelastic properties of both thermoplastic and thermosetting elastomers, effectively balancing the often‐conflicting requirements of structural rigidity and surface softness—critical for paediatric laryngeal stents. Our on‐demand design, material selection and manufacturing process are well‐suited for the rapid production of bespoke biocompatible implants with complex geometries, enabling a seamless transition from bench to bedside within clinically practical timeframes. The resulting custom stent contributed to excellent functional outcomes at an earlier age, with a reduced risk profile involving tracheostomy compared to conventional stenting options. This highlights the adaptability of composite design and advanced manufacturing techniques in creating patient‐specific medical devices that significantly improve both clinical outcomes and quality of life for patients with complex airway conditions.

## 6 Experimental Section

### Material Selection and Design

Medical grade thermoplastic polyurethane (TPU, Carbothane AC‐4085A‐B20) was purchased from Lubrizol. The Food and Drug Administration (FDA) approved medical grade silicones (NuSil MED‐4011 and MED4‐4420) were purchased from Silicone‐Polymer. N,N‐Dimethylacetamide (DMAc anhydrous, 99.8%) was purchased from Sigma–Aldrich.

### Manufacture of Interpenetrating Elastomer Network (IEN) Composites

In‐house 3D thermally induced phase separation (3D‐TIPS) process (i.e., inverse printing), as previously described, was adapted to fabricate TPU mesh scaffolds.^[^ ^15^, ^16^, ^17^ ^]^ Polyvinyl alcohol (PVA) preforms (50 mm × 10 mm × 2 mm) with varying infill densities (30% to 60%, in 10% increments) were printed using a Raise3D 3D printer. The TPU in DMAc solution was then injected into the PVA preforms through a surface puncture hole. These filled preforms were immersed in deionized ice water for 24 h, with regular water changes at room temperature, allowing the PVA to dissolve and the TPU to coagulate. The resulting TPU scaffolds were dried at room temperature and subsequently silicone resin was injected into the TPU scaffold using resin infusion moulding. Two PDMS formulations were used: MED‐4011 (viscosity≈97400 cP) and MED4‐4420 (viscosity≈23250 cP). Infusion was conducted under a vacuum of −80 kPa until visual observation confirmed complete transparency of the scaffold, indicating full pore filling. The infused composites were subsequently cured to yield TPU/PDMS IENs. Complete infiltration was further verified by cross‐sectional SEM imaging, which showed a continuous PDMS phase throughout the TPU network. The surface and cross‐sectional morphology of dried TPU scaffolds with various infill densities were examined using a digital camera and a field emission scanning electron microscope (FE‐SEM, Zeiss Sigma 300 VP, Germany).

### Mechanical Characterization of 3D TIPS Scaffolds and IEN Composites

The tensile mechanical properties of the TPU scaffolds with various infill densities and their corresponding IEN elastomer composites were evaluated using an Instron 5565 tester (Instron Ltd., USA) with a 500 N load cell. Tensile tests were conducted on samples with dimensions of 20 mm × 8.4 mm × 2 mm under uniaxial loading at 10 mm min^−1^. Force–displacement curves were recorded to calculate tensile modulus, ultimate tensile strength, strain at break, and toughness. The tensile modulus was determined as the slope of the linear fit of the stress‐strain curve over the first 15% strain. The ultimate strength was defined as the maximum stress, stretchability as the maximum strain, and toughness as the area under the stress–strain curve from initial deformation to rupture. Each measurement was performed with a minimum of four samples (n ≥4). The fracture surface of the composites was examined using the FE‐SEM (Zeiss Sigma 300 VP, Germany). The Shore A hardness of the composites was measured using a Shore Durometer.

### Biocompatibility Testing

To assess cytotoxicity, human lung fibroblasts (sourced with the informed consent of healthy non‐smoker donors from Lonza at passage 2) were seeded onto TPU scaffolds and TPU/PDMS composite discs (11 mm diameter; n = 6) at a density of 5 × 10^4^ cells cm^−3^ and cultured under standard culture conditions of 5% CO_2_ in incubator at 37.5 °C with medium renewal every 2–3 days. Wells containing 11 mm Thermonax coverslips (Nunc, USA) were used as tissue culture plastic (TCP) positive controls. The metabolic activity was monitored using an Alamar Blue fluorescence assay over seven days. The absorbance of the scaffolds and controls was measured by a fluorescence plate reader (Fluroskan Ascent FL reader, Thermo Labsystems, Basingstoke, UK) at 530 nm excitation wavelength and 620 nm emission wavelength. The cellular proliferation was monitored using a total DNA assay with a fluorescent Hoechst 33258 stain.

The in vivo biocompatibility of the composite stents was tested in an orthotopic New Zealand White (NZW) rabbit model of airway surgery.^[^ ^50^ ^]^ A 6 mm diameter tubular PDMS/TPU IEN composite stent was fabricated using a custom‐built rotational 3D printer, in line with NZW rabbit tracheal dimensions, which are highly conserved for a given animal's weight.^[^ ^51^ ^]^ Under Home Office Licence 70/7504, New Zealand white rabbits of 2.5–3.5 kg weight underwent open tracheotomy under general anaesthesia with recovery, and 1 cm lengths of either PDMS/TPU IEN composite (*n* = 4) or standard endotracheal tube (*n* = 4) were inserted into the mid‐portion of the trachea. These were secured in place with a through‐and‐through suture to prevent migration as per laryngotracheal reconstruction clinical practice. Animals were sacrificed after two weeks, in line with the intended length of clinical use, and the tracheae were explanted adjacent to the stent for histological analysis.

### Laryngeal Stent Design and Manufacture for Clinical Use—Institutional Approvals

Following verification of the composite stent's mechanical performance and favorable in vivo airway biocompatibility, approval for a non‐CE‐marked custom design and stent fabrication process was obtained from the UK Medicines and Healthcare products Regulatory Agency on humanitarian grounds (HAL/2017/053). Additionally, the GOSH Clinical Ethics Committee granted a favorable opinion for its use in May 2017.

### Laryngeal Stent Design and Manufacture for Clinical Use—Fabrication and Characterization of Custom Composite Laryngeal Stent

The custom stent was designed using CAD based on measurements taken during MLB to match achievable target neolaryngeal dimensions. A PVA preform of the laryngeal stent was then 3D‐printed with a 50% infill density. The scaffold structure was created by injecting a TPU DMAc solution (LUBRIZOL AC‐4085A Carbothane, DMS) into the mould and processing it with cryo‐coagulation (3D‐TIPS), resulting in a porous TPU framework. Silicone resin (MED‐4011, Dow Corning, FDA medical grade) was then infused into this TPU scaffold using a resin infusion method, forming the final IEN composite laryngeal stent.

Static compression tests were conducted using an Instron 5565 tester at a compressive speed of 5 mm min^−1^. Compressive stiffness was determined by the slope of the linear region of the force–displacement curve for the first 1 mm elongation. The dynamic mechanical properties of the bespoke laryngeal stent were evaluated using a sinusoidal ramp at 1 Hz and controlled compressive displacement of 1 mm for 20 000 cycles, using a dynamic mechanical tester (ElectroForce 5160, TA Instrument, USA) with a 200 N load cell.

### Laryngeal Stent Design and Manufacture for Clinical Use—GMP Standard Manufacture and Sterilization

The composite stent was classified as a Class IIa medical device. The GMP manufacturing process for the EIN stent, made from medical‐grade silicone and polyurethane, involved using high‐purity materials from verified suppliers, conducting production in ISO Class 7 or 8 cleanrooms with continuous environmental monitoring, and validating all equipment and processes. In‐process testing, assembly in clean environments, validated packaging for sterility, and maintaining detailed batch records for full traceability were implemented to ensure compliance with GMP standards. The sterilization and microbiological assessments were provided by Hunt Development Ltd.

### Statistical Analysis

Data are presented as mean ± SD. Statistical analysis was performed using GraphPad Prism 10 software, and the statistical methods used are stated in the figure legends. Mostly, multiple groups were typically compared using one‐way ANOVA with Tukey's post‐hoc test, while comparisons between two groups were usually conducted using the t‐test, with *p* <0.05 considered statistically significant.

## Conflict of Interest

The authors declare no conflict of interest.

## Author Contributions

E.F.M. and L.W. contributed equally to this work. E.F.M., T.K., R.H., and W.S. conceptualized the study. L.W., D.Z., E.F.M., L.W., G.M., H.M., and W.J.T. carried out design, fabrication, and characterization of the materials and stents. GMP fabrication of stent was carried out by D.Z., L.W., T.J.P., M.W.L., and M.A.B. Surgery was performed by E.F.M., C.R.B., and R.H. Writing original draft: E.F.M., L.W., and W.S. wrote the original draft. All the authors wrote, reviewed, and edited the final manuscript.

## Supporting information



Supporting Information

## Data Availability

The data that support the findings of this study are available on request from the corresponding author. The data are not publicly available due to privacy or ethical restrictions.
