# Supplementary material for: A Bespoke Programmable Interpenetrating Elastomer Network Composite Laryngeal Stent for Expedited Paediatric Laryngotracheal Reconstruction
Source: Adv Funct Mater. 2025 Nov 10;36(24):e13861. doi: 10.1002/adfm.202513861 (PMC13709330; doi:10.1002/adfm.202513861)
Supplement: Supplementary file 1 [file ADFM-36-e13861-s001.docx]

# Supporting Information

**A bespoke programmable interpenetrated elastomer network composite laryngeal stent for expedited paediatric laryngotracheal reconstruction**

Elizabeth F. Maughan^†^*, Lei Wu^†^, Dong Zhang, Linxiao Wu, Graca Ferreira Vaz Pinto Mendes^4^, Wanqiao Tu, Heping Ma, Toby J. Procto^5^, Colin R. Butler, Thomas Kjærgaard, Mark W. Lowdell, Martin A. Birchall_,_ Robert E. Hynds, Richard Hewitt and Wenhui Song*

Figure S1. Comparisons of mechanical and anatomical parameters and manufacturing methods of the bespoke IEN stent versus existing laryngeal stents (LT-Mold^TM^ and Rutter stent).

| Table S1 Properties of Medical Grade elastomers | | | | | |  | |  | |  |  |
| --- | --- | --- | --- | --- | --- | --- | --- | --- | --- | --- | --- |
| Material | Series | Mixed Viscosity (cP) | Shore Hardness | Tensile strength (MPa) | Tensile Strain at Break (%) | | Tensile Modulus (MPa) | | References | |  |
| Silicone | NUSIL  MED-6233 | 73500 | 50A | 5.1 | 380 | | 4.5 | | Avantor Inc. | |  |
|  | NUSIL  MED-4011 | 97400 | 25A | 4.7 | 530 | | 0.9 | | Avantor Inc. | |  |
|  | NUSIL  MED4-4420 | 23250 | 17A | 4.8 | 600 | | 0.6 | | Avantor Inc. | |  |
| TPU | LUBRIZOL  AC-4085A | N/A | 85A | 55 | 400 | | 6.03 | | Lubrizol Corporation | |  |
|  | LUBRIZOL  AC-4095A | N/A | 95A | 68.9 | 370 | | 14.6 | | Lubrizol Corporation | |  |
| Adult | Thyroid/Tracheal cartilage |  | 59.61-91 Shore A | 1-15 MPa |  | | 3.2-23 MPa;  12.2±1.3 MPa (Young, 18-36 years)  20.5±1.8MPa (Old, 49-65 years) | | Rains, 1992^[19]^; Sicard,  2018^[20]^  Safshekan  2017^[44]^ | |  |
| Adult | Supraglottic Epiglottis |  | ~20-30 Shore A (estimated) | ~1.5 MPa (auricular elastic cartilage, estimated) |  | | ~0.29 MPa | | Brown, 2013^[49]^;  Pauken, 2018^[50]^;  Grellmann, 2006^[21]^ | |  |
| Adult | Vocal fold |  | ~5-15 Shore 00 (estimated) | ~0.35-1.75 MPa |  | | ~0.03 MPa | | Alipour, 2012^[15]^ | |  |
| Rabbit* | Airway mucosal membrane (tracheal/subglottic) |  |  |  |  | | 4-18 kPa  6.5±4.0 kPa (Circumferential);  12.8±6.6kPa (Longitudinal) | | Wang, 2000^[51]^ | |  |

* Female New Zealand White rabbits between 0.75 and 35 months of age and the youngest group of animals (between 0.75 and 1 month old). The tests reported that the airway mucosal membrane was stiffer in the longitudinal than in the circumferential direction of the airway. However, there was no signiﬁcant change with age in either orientation.
